# Knowledge and utilisation of National Health Insurance Scheme among adult patients attending a tertiary health facility in Lagos State, South-Western Nigeria

**DOI:** 10.4102/phcfm.v11i1.2018

**Published:** 2019-09-04

**Authors:** Abdulhakeem O. Abiola, Temitope W. Ladi-Akinyemi, Oluwagbemiga A. Oyeleye, Ganiyat K. Oyeleke, Olufunke I. Olowoselu, Aisha T. Abdulkareem

**Affiliations:** 1 Department of Community Health and Primary Care, College of Medicine, University of Lagos, Lagos, Nigeria; 2 Department of Medicine, Lagos University Teaching Hospital, Lagos, Nigeria; 3 Department of Community Health, Lagos University Teaching Hospital, Lagos, Nigeria

**Keywords:** National Health Insurance Scheme, Lagos State University Teaching Hospital, LASUTH, patients, adults, knowledge of NHIS, utilisation of NHIS

## Abstract

**Background:**

Despite the creation of the National Health Insurance Scheme (NHIS) by the Nigerian government, most Nigerians are not covered by the scheme.

**Aim:**

The aim of this study was to assess the knowledge and utilisation of NHIS among adult patients who attended a tertiary health facility in Lagos state, South-Western Nigeria.

**Setting:**

Outpatient clinic, Lagos State University Teaching Hospital, Ikeja, Lagos.

**Methods:**

A descriptive cross-sectional study of 487 respondents recruited using a multi- stage sampling method. Data were collected using pretested semi-structured self-administered questionnaires, and analysis was done using Microsoft Excel 2007 and EPI Info 7 statistical software. Level of significance was set at *p* < 0.05. Ethical approval was obtained from the Health Research Ethics Committee Lagos State university teaching Hospital.

**Results:**

A total of 487 of the 500 self-administered questionnaires were retrieved and analysed, giving a response rate of 97.4%. The study showed that 80.7% of the respondents had poor knowledge of NHIS, only12.3% of the respondents had registered with the NHIS, and 43.8% of respondents who had not registered with NHIS claimed they do not know where to register. There was a statistically significant association between age and utilisation (*p* = 0.0007), marital status and utilisation of NHIS (*p* = 0.016), employment status and utilisation of NHIS (*p *= 0.001).Most (96%) of those who have utilised NHIS were satisfied with NHIS services.

**Conclusion:**

Majority of the respondents had poor knowledge of NHIS and also majority of those who had registered were satisfied with the scheme. There should be increased awareness campaigns so that all Nigerians can benefit from the scheme.

## Introduction

The National Health Insurance Scheme (NHIS) is a type of formal sector social health insurance programme.^1^ In this programme, the cost of health care for an employee is paid for by the contributions of both the employee and the employer, which makes it a social health security system.^1^ This is made possible by deductions of 5% of the employee’s salary and 10% of the employer’s salary paid out to the employee on a monthly basis, the whole resources are then used to cover the health expenditures of all those enrolled under the scheme.^2^ The concept also involves that of subsidisation in which the young subsidise for the old, the rich subsidise for the poor and the healthy subsidises for the ill.^2^

The National Health Insurance Scheme aims at ensuring that every Nigerian has access to good healthcare services, protecting families from the financial hardship of huge medical bills.^1^ It also limits the rise in the cost of healthcare services, thereby maintaining high standard of healthcare delivery services within the system.^1^ It ensures both the efficiency in healthcare services and the availability of funds to the health sector for improved services, which guarantees equitable patronage of all of health care.^1^ The NHIS is seen as a complementary means of financing health care that is, to the government, international organisation and the whole community, a major factor necessary for the aim of achieving a worldwide level of good health that will lead to a productive and socially stable economy.^3^

The benefits of implementing the Compulsory Health Insurance Scheme includes three aspects: the scheme provides for the pooling of resources for cross subsidisation of health costs where those in high-income bracket subsidises those in low income bracket^1^; the healthy subsidise the sick and the young subsidise the old^1^; the burden of funding healthcare services is shared between government and private employers and enrollees.^1^ Quackery is also minimised owing to standards for personnel and equipment set for providers by the scheme.^1^ Competition among healthcare providers to attract and retain clients leads to improvement in the quality of services.^1^ The scheme provides employment opportunities for health professionals in the healthcare delivery system and thereby reduces brain drain.^3^ Donor Agencies and/or government will have the confidence to donate to rural communities or less privileged through the proposed vulnerable group fund.^3^ Improved services resulting from improved income reduces the need for overseas treatment, thereby conserving the country’s foreign reserve.^3^ Improved access to health care services create a healthier work force for increased economic activities and national prosperity.^3^ Communities and associations contribute for their health care services without viewing it as tax or levy, as they are the financial managers and administrators of their programme and reap the benefits from there.^3^

Members who are enrolled in this scheme get to enjoy several benefits and services such as hospital care (limited to only 15 days in a year and admission in the general ward), outpatient care, pharmaceutical care as in NHIS essential drug list, diagnostic tests as in NHIS diagnostic test list, maternal care for up to four (4) life births; preventive care (immunisation, health education, antenatal and postnatal care), eye care and preventive dental care.^2^ Beneficiaries no longer need to pay for treatment with cash in health centres when required except for the 10% co-payment for the price of drugs.^2^ This will reduce or even totally eliminate the usual habit of family members selling off their assets in order to generate money for treatment in emergency cases of life threatening conditions and illnesses. According to the ministry of health, it claims that the National Health Insurance Scheme is in fact the most comprehensive in the world owing to its benefit package.^2^

However, Nigeria remains the country with the highest number of out-of-pocket payments for healthcare, yet the health system is still very poor.^4^ The poor state of the health system was what led to the initiation and establishment of the National Health Insurance Scheme by the Nigerian Federal State.^4^ In 1997, its policy was drafted by the Nigerian government, which was then signed into law in 1999 and was launched for implementation on 16th June 2005.^4^ People die daily from common illnesses, which can be easily treated owing to financial incapacity.^5^ Many individuals borrow or loan money, sell their properties, use up all of their personal savings just to ensure they can pay for their health care.^6,7^

The major objective of the NHIS was to ensure that it covers all indigent Nigerians across the country with the purpose of delivering a comprehensive and affordable healthcare nationally. It comprises employers, employees, self-employed, unemployed and rural communities.^2^ The Nigerian health insurance scheme only covers 4% of the population, which is significantly low.^8^ Nine states have indicated interest in the scheme (Abia, Enugu, Gombe, Imo, Jigawa, Kaduna, Lagos, Ondo and Oyo) but only two states (Bauchi and Cross-river) attempted to enrol their employees in the scheme.^9^ The aim of this study was to identify the problems associated with the utilisation of the health insurance scheme.

The specific objectives of this study were to assess the knowledge of health insurance among adult patients to find out the degree of utilisation of health insurance among these patients and to determine some of the factors that affect the utilisation of health insurance among these patients in the Lagos State University Teaching Hospital, Ikeja.

## Methodology

### Description of study area

The study was conducted in the adult outpatient department of the Lagos State University Teaching Hospital (LASUTH), formerly known as Ikeja General Hospital. The Lagos State University Teaching Hospital is a tertiary health institution situated in Ikeja, Local Government Area of Lagos State in South-western Nigeria. It is a multi-disciplinary tertiary hospital and it has a total bed space of 520. It is a major referral centre serving the whole of Lagos State, which is the economic nerve centre of Nigeria. It has many specialists’ clinics and runs 24 hours-accident and emergency services and inpatient care services.^10^ Although the hospital is a tertiary healthcare facility, it also serves as a primary and secondary healthcare facility; hence, its services are affordable. Consultation and other services are rendered free of charge and only prescribed medicine and laboratory investigations are paid for by the patients.

### Methods

The study was a descriptive cross-sectional study assessing the knowledge, attitude and the utilisation of NHIS among adult patients who were 18 years and above, attending the outpatient clinics of Lagos State University Teaching Hospital (LASUTH). The minimum sample size required for the study was approximately 465 using the Cochrane formula for cross-sectional study:

$$n={\text{Z}}^{2}\text{p q}/{\text{d}}^{2}$$ [Eqn 1]

where *n* = minimum sample size for a target population ≥ 10 000, Z = standard normal deviate corresponding to 95% confidence interval, *p* = prevalence of attitude towards health insurance from a previous study, *q* = 1-p and *d* = degree of accuracy desired. The respondents were recruited using a multi-stage sampling method. In stage one: 10 of the 16 outpatient clinics present in LASUTH were selected by simple random sampling method using balloting procedure. In stage two, systematic sampling method was used to select respondents in the selected clinics.

The number of clients selected in each clinic was determined by proportionate allocation based on the statistics obtained from the administrative department.

### Inclusion criteria

Patients aged 18 years and above and also attending any of the outpatient clinics in LASUTH.

### Exclusion criteria

Patients who were unconscious or had mental illness

### Data collection method

Data were collected from the respondents in the clinics using a pretested, semi-structured self-administered questionnaire. The questionnaire was divided into three sections: (1) Section A: Socio-demographic characteristics of the respondents such as age, sex, tribe, level of education, employment status, (2) Section B: Knowledge about National Health Insurance Scheme and (3) Section C: Utilisation of National Health Insurance Scheme.

### Data analysis

Data were analysed using Microsoft Excel 2010 and EPI info software version 7, a public domain software developed by Centre for Disease Control and prevention (CDC). Data were summarised using means, standard deviation and proportions. The data were presented using tables. Relationships between categorical variables were tested using the Chi-square test and Fisher’s exact test. Relationships between continuous variable were tested using the student *t*-test *p*-value ≤ 0.05 was considered statistically significant.

### Scoring system

The knowledge questions were scored and the overall grade was ascertained. One point was awarded for each correct answer given to the questions and no point was awarded for incorrect or ‘I don’t know’ responses.

Knowledge had a total score of 11 and knowledge was graded as either poor (score of 0–6) or good (score of 7–11).

### Ethical considerations

Ethical approval was obtained from the Health Research Ethics Committee of LASUTH (LREC. 06/10/912). Verbal permission for the study was obtained from the Doctors and Matrons on duty in the outpatient clinics. Informed consent was also obtained from each respondent. The confidentiality of information collected was secured by restricting access to the data collected to investigator and research assistants. Anonymity of the respondents was ensured by not including the personal details of the respondents in the instrument. Respondents were assured that their responses will not be used against them and it will not influence the care they will receive in the facility.

## Results

A total of 487 of the 500 administered questionnaires were retrieved and analysed giving a response rate of 97.4%. The mean age of the respondents was 34.1 ± 12.1 years. Among the respondents, 35.3% were in the age range of 21–30 years, 56.7% were married, 54.0% were females, 65.5% were Christians, 58.7% had tertiary education, and 32.7% were civil servants (Table 1).

**TABLE 1:** Socio-demographic characteristics of the respondents (*n* = 487).

Variables	Frequency	Percentage
**Age (years)**		
> 18	57	11.7
21–30	172	35.3
31–40	140	28.8
41–50	72	14.8
51 and above	46	9.4
Mean ± s.d.	34.1 ± 12.1	-
**Sex**		
Male	224	46.0
Female	263	54.0
**Religion**		
Christian	319	65.5
Muslim	157	32.2
Others	11	2.3
**Marital status**		
Single	184	37.8
Married	276	56.7
Divorced/Separated/widow	27	5.5
**Highest level of education**		
No formal education	15	3.1
Primary education	20	4.1
Secondary education	166	34.1
Tertiary education	286	58.7
**Employment status**		
Civil servant	159	32.7
Private sector	104	21.4
Self-employed	134	27.5
Student	54	11.1
Unemployed	36	7.3

s.d., standard deviation.

## Knowledge

Only 7.2% of the respondents knew that NHIS does not provide maternity care for more than four live births for every insured person. Eleven point one percent (11.1%) of the respondents knew the total monthly contribution by both the formal sector employee and employer, and 13.6% of the respondents knew the percentage of the employee’s wages to be contributed by the employee (Table 2).

**TABLE 2:** Respondents’ knowledge of National Health Insurance Scheme (*n*=487).

Knowledge of NHIS	Correct response	
	Frequency	Percentage
The NHIS is based on the concept of social health insurance	252	51.8
The NHIS aims to ensure that every Nigerian has access to good health care services	348	71.7
The NHIS does not provide maternity care for up to five (5) live births for every insured person	35	7.2
The NHIS implementation is in phases, starting with employees in the formal sector (public and private)	250	51.3
The NHIS makes provision for vulnerable groups	73	15.0
A contribution made by the insured person entitles him or herself, spouse and four (4) children under the age 18 years	241	49.5
The total monthly contribution by both the formal sector employee and employer is not 20% of his or her wages	54	11.1
The employee is to pay 5% of his or her wages	66	13.6
Health Maintenance Organisations are the financial managers of NHIS	124	25.5
Capitation is the sum of money paid monthly by the HMOs to healthcare providers on behalf of a contributor whether or not services are rendered by the healthcare provider	111	22.8
An insured person pays 10% of the actual cost of drugs	72	14.9

NHIS, National Health Insurance Scheme, HMOs, health maintenance organisations.

## Utilisation

Most (87.7%) of the respondents have not registered with the NHIS and 43.8% of those who have not registered did not know where to register. Most (83.3%) of the respondents who had registered with NHIS had utilised the scheme for treatment in the last 6 months. Only 10% of those who have registered with NHIS have utilised it for surgical treatment. Majority (96.0%) of those who had utilised the NHIS claimed that they are satisfied with the healthcare services provided by the scheme (Table 3).

**TABLE 3:** Respondents’ utilisation of National Health Insurance Scheme.

Variables	Frequency	Percentage
**Have you registered with NHIS (*n* = 487)**		
Yes	60	12.3
No	427	87.7
**If you have not registered with NHIS, state the reasons why you have not registered (*n* = 427)**		
I don’t know where to register	187	43.8
I am not interested in NHIS	71	16.6
I do not have time to register	66	15.5
Others	103	24.1
**Have you obtained your NHIS card (*n* = 60)**		
Yes	47	78.3
No	13	21.7
**Have you utilised NHIS for treatment in the last 6 months (*n* = 60)**		
Yes	50	83.3
No	10	16.7
**Have you utilised the NHIS for Surgery (*n* = 60)**		
Yes	6	10.0
No	54	90.0
**Are you satisfied with NHIS services (*n* = 50)**		
Yes	48	96.0
No	1	2.0
I don’t know	1	2.0

NHIS, National Health Insurance Scheme.

## Factors affecting utilisation of health insurance

Table 4 looks at variables associated with knowledge of NHIS and shows that those with education were more likely to have better knowledge of NHIS and to register with the scheme. Table 5 looks at variables associated with registration with NHIS and shows that those who were older registered more because they were at risk of more diseases. Table 6 also looks at variables associated with utilisation of NHIS and shows that those who were married utilised the services of NHIS more because it covers an individual, a spouse and four family members.

**TABLE 4:** Factors affecting knowledge of National Health Insurance Scheme among respondents (*n* = 487).

Variables	Knowledge of NHIS		*p*
	Poor, *n* = 393	Good, *n* = 94	
**Age (years)**			0.160
Mean ± s.d.	2.5 ± 1.9	6.7 ± 1.0	
**Sex**			0.166
Male			
Frequency (n)	185	39	
Frequency (%)	47.1	41.5	
Female			
Frequency (n)	208	55	
Frequency (%)	52.9	58.5	
**Religion**			0.112*
Christian			
Frequency (n)	249	70	
Frequency (%)	63.4	74.5	
Muslim			
Frequency (n)	135	22	
Frequency (%)	34.4	23.4	
Others			
Frequency (n)	9	2	
Frequency (%)	2.2	2.1	
**Marital status**			0.321*
Single			
Frequency (n)	152	32	
Frequency (%)	38.7	34.0	
Married			
Frequency (n)	216	59	
Frequency (%)	55.0	62.8	
Divorced/separated/widow			
Frequency (n)	25	3	
Frequency (%)	6.3	3.2	
**Highest level of education**			0.050*
No formal education			
Frequency (n)	13	2	
Frequency (%)	3.3	2.1	
Primary			
Frequency (n)	20	0	
Frequency (%)	5.1	0.0	
Secondary			
Frequency (n)	137	29	
Frequency (%)	34.9	30.9	
Tertiary			
Frequency (n)	223	63	
Frequency (%)	56.7	67.0	
**Employment status**			0.208
Civil servant			
Frequency (n)	121	38	
Frequency (%)	30.8	40.4)	
Private sector			
Frequency (n)	88	16	
Frequency (%)	22.4	17.0	
Self-employed			
Frequency (n)	106	28	
Frequency (%)	27.0	29.8	
Student			
Frequency (n)	48	6	
Frequency (%)	12.2	6.4	
Unemployed			
Frequency (n)	30	6	
Frequency (%)	7.6	6.4	
**NHIS registration**			0.023*
Yes			
Frequency (n)	42	18	
Frequency (%)	10.7	19.1	
No			
Frequency (n)	351	76	
Frequency (%)	89.3	80.9	

NHIS, National Health Insurance Scheme; s.d., standard deviation.

* , Fisher’s exact.

**TABLE 5:** Factors affecting registration with National Health Insurance Scheme (*n* = 487).

Variables	Registration with NHIS		*p*
	Yes, *n* = 60	No, *n* = 427	
**Age (years)**			0.001
Mean ±s.d.	38.9 ± 14.5	33.5 ± 11.6	
**Sex**			0.239
Male	25	199	
Frequency (%)	41.7	46.6	
Female	35	228	
Frequency (%)	58.3	53.4	
**Religion**			0.711*
Christian	40	279	
Frequency (%)	66.7	65.3	
Muslim	18	139	
Frequency (%)	30.0	32.6	
Others	2	9	
Frequency (%)	3.3	2.1	
**Marital status**			0.169*
Single	19	165	
Frequency (%)	31.7	38.6	
Married	40	236	
Frequency (%)	66.7	55.3	
Divorced/separated/widow	1	26	
Frequency (%)	1.6	6.1	
**Highest level of education**			0.273*
No formal education	0	15	
Frequency (%)	0.0	3.5	
Primary	1	19	
Frequency (%)	1.7	4.4	
Secondary	18	148	
Frequency (%)	30.0	34.7	
Tertiary	41	245	
Frequency (%)	68.3	57.4	
**Employment status**			0.001*
Civil servant	24	135	
Frequency (%)	40.0	31.6	
Private sector	20	84	
Frequency (%)	33.3	19.7	
Self-employed	1	133	
Frequency (%)	1.7	31.1	
Student	13	41	
Frequency (%)	21.7	9.6	
Unemployed	2	34	
Frequency (%)	3.3	8.0	

**TABLE 6:** Factors affecting utilisation of National Health Insurance Scheme among respondents (*n* = 487).

Variables	Registration with NHIS		*p*
	Yes, *n* = 60	No, *n* = 427	
**Age (years)**			0.0007
Mean ±s.d.	39.6 ± 12.3	33.5 ± 11.9	
**Sex**			0.369
Male	20	204	
Frequency (%)	40.0	46.7	
Female	35	233	
Frequency (%)	58.3	53.3	
**Religion**			0.435*
Christian	34	285	
Frequency (%)	68.0	65.2	
Muslim	14()	143	
Frequency (%)	28.0	32.7	
Others	2	9	
Frequency (%)	4.0	2.1	
**Marital status**			0.016*
Single	13	171	
Frequency (%)	26.0	39.1	
Married	37	239	
Frequency (%)	74.0	54.7	
Divorced/separated/widow	0	27	
Frequency (%)	0.0	6.2	
**Highest level of education**			0.483*
No formal education	0	15	
Frequency (%)	0.0	3.4	
Primary	1	19	
Frequency (%)	2.0	4.3	
Secondary	15	151	
Frequency (%)	30.0	34.6	
Tertiary	34	252	
Frequency (%)	68.0	57.7	
**Employment status**			0.001*
Civil servant	20	139	
Frequency (%)	40.0	31.8	
Private sector	16	88	
Frequency (%)	32.0	20.1	
Self-employed	1	133	
Frequency (%)	2.0	30.4	
Student	11	43	
Frequency (%)	22.0	9.8	
Unemployed	2	34	
Frequency (%)	4.0	7.9	

## Discussion

More than one-third of the respondents were between 21 and 30 years’ age range with a mean age of 34.1 (12.1 standard deviation [s.d.]). This is in contrast to a similar study conducted in rural southwest of Nigeria where about 69% of the respondents belong to the age range of 24–45 with the mean age of 39 (7.12 s.d.).^4^ The study also revealed that more than half of the respondents were married, which means that the NHIS scheme is expected to get more populated because a married individual can also register other members of the family into the scheme. More than half of the respondents had tertiary education, which was in contrast with the findings from the study in the rural southwest of Nigeria, where most of the respondents (63.2%) were Primary/secondary school leavers. This may be because this study was conducted in a tertiary health facility in Lagos state.

Furthermore, the results of this study revealed that less than one-third of the respondents have good knowledge of NHIS, which is consistent with findings from a similar study carried out in Lagos state among primary healthcare managers in which 17.9% had good knowledge^11^and another study among dentists which showed that only 28.7% had good knowledge.^12^ This is strange because it is expected that medical and dental practitioners should have good knowledge of NHIS. The study also revealed that over two-thirds of the respondents knew the objectives of NHIS; this is in contrast to a similar study where only 38.8% knew the objectives,^13^ and also another study where only 26.7% knew the objectives.^14^

According to this study, less than one-fifth of the respondents have registered with the NHIS, which is similar to the study carried out in secondary health facilities in Lagos state, where only 10.1% of the respondents registered with NHIS.^15^ However, the findings of this study is higher than that of a similar study in Osun state, where only 0.3% of respondents had registered with the scheme and another where just one respondent out of 4873 respondents was registered.^13^ Although a similar study in Oyo state showed that 83.2% of the respondents who were majorly civil servants had registered with the NHIS because it was made compulsory among government workers in the formal sector, but only 58.9% had started utilising the services.^16^ In another study among University workers, only 49.8% claimed they had registered with the scheme.^17^

In this study, over three-quarters of those who registered have obtained their NHIS cards. It was found out that most of those registered rarely used the NHIS for surgical services. This may be because most of the surgical procedures are not covered by the NHIS and patient has to pay out-of-pocket for such procedures. Almost half of the respondents used NHIS to treat medical ailment, which is similar to a study carried out in Ibadan where 52.9% of the respondents utilised the NHIS to also treat medical ailments such as Malaria.^18^ It was also found out that a very high percentage of the respondents were satisfied with the healthcare services provided by the NHIS. This finding was higher than that of a similar study in Oyo state where about 60% of the respondents were also satisfied with the scheme.^16^

Respondents who had attained tertiary level of education had more knowledge of NHIS and therefore registered with the scheme compared with respondents who had a lower level of education. In addition, it was also found out that age and employment status are determining factors for registration with the NHIS. Majority of the respondents who were civil servants registered with the NHIS as it is made mandatory by the government for this category of workers. Furthermore, respondents who were married utilised the NHIS services more because the scheme covers a registered member, a spouse and four children. Older respondents tend to have increased morbidity; hence they require the services of NHIS than respondents who are younger and healthy.

In summary, the study demonstrates that majority of the respondents have poor knowledge of NHIS. Majority of the respondents have not registered with the NHIS and their excuse was that they did not know where to register. A large percentage of the respondents who had registered claimed they are satisfied with the services provided by the scheme. There were significant association between age, and employment status of the respondents and their registration and utilisation of NHIS.

## Limitations

A potential bias in this study was recall. However, recall bias was reduced by limiting enquiries on utilisation of NHIS to the last 6 months. This may tend to underestimate the level of utilisation. Another limitation was that the implementation of the NHIS outside of the original intentions and policy framework accounted for the poor utilisation measured. Also, this is a facility-based study and the results cannot be generalised to the general population.

## Conclusion

Majority of the respondents involved in this study had poor knowledge of the NHIS, but majority of those who had registered were satisfied with the services of the NHIS. Those who had not registered with the NHIS claimed they do not know where to register. There should be increased awareness campaigns on all forms of media with emphasis on locations of registration centres and a step-by-step process of how to register with the scheme. The government should ensure that the NHIS is mandatory for both the formal and informal sector employee so that all Nigerians can benefit from the scheme.
